# Perceived similarity as a window into representations of integrated sentence meaning

**DOI:** 10.3758/s13428-023-02129-x

**Published:** 2023-06-22

**Authors:** Sophie Arana, Peter Hagoort, Jan-Mathijs Schoffelen, Milena Rabovsky

**Affiliations:** 1 Max Planck Institute for Psycholinguistics, Nijmegen, The Netherlands; 2 Donders Institute for Brain, Cognition and Behaviour, Radboud University, Nijmegen, The Netherlands; 3 Department of Psychology, University of Potsdam, Potsdam, Germany

**Keywords:** Similarity, Semantics, Thematic roles, Multiple arrangement task, Factorization

## Abstract

When perceiving the world around us, we are constantly integrating pieces of information. The integrated experience consists of more than just the sum of its parts. For example, visual scenes are defined by a collection of objects as well as the spatial relations amongst them and sentence meaning is computed based on individual word semantic but also syntactic configuration. Having quantitative models of such integrated representations can help evaluate cognitive models of both language and scene perception. Here, we focus on language, and use a behavioral measure of perceived similarity as an approximation of integrated meaning representations. We collected similarity judgments of 200 subjects rating nouns or transitive sentences through an online multiple arrangement task. We find that perceived similarity between sentences is most strongly modulated by the semantic action category of the main verb. In addition, we show how non-negative matrix factorization of similarity judgment data can reveal multiple underlying dimensions reflecting both semantic as well as relational role information. Finally, we provide an example of how similarity judgments on sentence stimuli can serve as a point of comparison for artificial neural networks models (ANNs) by comparing our behavioral data against sentence similarity extracted from three state-of-the-art ANNs. Overall, our method combining the multiple arrangement task on sentence stimuli with matrix factorization can capture relational information emerging from integration of multiple words in a sentence even in the presence of strong focus on the verb.

## Introduction

We perceive the world around us sequentially, a few bits of information at a time (e.g., saccade by saccade, word by word), but we rarely ponder each bit of information in isolation (apples, bowl, table). Instead, we perceive entire scenes, within which relations between objects are inherent (apples IN bowls AND bowls ON tables) (Hafri & Firestone, 2021). Similarly, when reading a sentence, we quickly integrate each word into the larger sentence meaning, by taking into account both semantic as well as structural information.

We can process isolated words based on their word semantics. For example, we can categorize them purely based on their semantic category (e.g., tool, profession). Nouns from the same professional domain such as “doctor and nurse” will be perceived as more similar to each other, compared to nouns sampled from different professional domains such as “doctor and carpenter”, although perceived similarity might change depending on the wider context. Here we refer to word semantics as the semantic categories relevant in a given context.

The meaning of a sentence is dependent on multiple words and hence might stretch across multiple semantic categories. For example, two sentences may both share nouns from similar professional domains, but describe very different events due to the verb semantics, i.e., the semantic category of the verb. For example, the doctor praises the nurse is perceived as very different from the nurse scolds the doctor. Additionally, a complete understanding of a sentence involves monitoring for structural information, e.g., expressed through word order. Structural cues allow us to infer crucial information about thematic role assignment, i.e., who the agent and patient of an event are. In the example given above, the nurse is a receiver of praise in the first proposition but is the person delivering scold in the second. Such thematic role information is abstract, i.e., we can flexibly apply it to new or unusual exemplars and complete reversal of roles can change the propositional content in a profound way (e.g., “the dog bit the old lady” vs. “the old lady bit the dog”). Sentence comprehension thus relies on multiple streams of information including the semantic categories of content words and structural information such as thematic role assignment.

There is an ongoing debate in cognitive neuroscience as to how integrated meaning combining both semantic as well as relational information is processed and represented (Rabovsky & McClelland, 2020; Puebla, Martin, & Doumas 2021). Empirical approaches to investigating integration of meaning in multi-word utterances require quantitative measures of sentence meaning. Past studies have relied on distributional semantics (Lyu et al., 2019) or constructed minimal contrasts between sentences with shared or no shared structure (Frankland & Greene, 2020b). In this study, we explore the utility of empirical, perceived similarity judgments as quantitative models of integrated sentence meaning. Specifically, we focus on whether they can capture relational information.

### Similarity as a window into sentence representations

Similarity is a useful concept to investigate representational content, when explicit quantitative models of a stimulus are not available. The underlying assumption when asking experimental subjects to make explicit similarity judgments in the lab under controlled conditions is that those judgments presumably reflect some aspects of people’s mental representations of the judged items at that instance. For example, two sentences might differ in certain features such as a single word or a thematic role assignment but are judged as highly similar. This response would suggest that the discrepant feature was not part of the reader’s mental representation of that sentence when making the judgement.

While similarity judgments have been used to quantify representational content in the past, in the domain of language, this has been mostly limited to quantifying single-word meaning, and so similarity has not yet been shown to capture more complex propositional meaning such as thematic role assignment, which emerges from the interaction of multiple linguistic cues. There are reasons to believe, however, that similarity judgments can capture structural information beyond single words. Previous studies on visual scene perception indicate that relational information might indeed influence perceived similarity. For example, participant’s judgment of scene similarity varied more strongly when the diverging feature was structurally aligned across scenes, i.e., a change in an existing element can be big or small, as compared to when it was not structurally aligned, i.e., adding a new element creates a difference independent of its exact features (Markman & Gentner, 1996). Sentences, just like visual scenes, include structural information, but unlike scenes may be perceived as more fragmented due to separation between words.

### Context effects create bias in similarity ratings

While in principle, many aspects of a sentence may influence the representation it evokes in the receiver’s mind, in practice only a few of those aspects will be dominant in any given situation. Similarity judgments can change depending on which feature is diagnostic in a given context, i.e., a feature that allows to differentiate some items from the others would be diagnostic, whereas a feature that is shared across all items would not be diagnostic (Tversky, 1977). When asking to judge a sentence’s similarity in relation to other sentences, the stimulus set provides a task context, which influences the degree to which features will be taken into account when judging each pairwise similarity. For example, if a set of 20 sentences varied in two dimensions, e.g., the semantic categories of the nouns and verb that appear in the sentences, the dimension with less variability may be recognized first as a common denominator between sentence pairs and hence be chosen as the diagnostic feature that drives similarity judgments. If there were fewer verb semantic categories, for example, the verb semantics could become the diagnostic feature which would lead to a behavioral bias to judge sentences based on the verb more so than on the nouns they contain. If this bias is strong enough, it might lead us to conclude that the noun semantics do not modulate the mental representation of the sentences in this task. Therefore, the conditions under which similarity judgments were collected always need to be carefully evaluated with respect to any conclusions drawn from them.

Even during naturalistic processing, however, attentional bias can be induced by a variety of linguistic (e.g., word meaning, word order, morphological markers) and extra-linguistic cues (concomitant gesture and eye gaze). For example, natural language processing relies heavily on information structure, i.e., some words are often emphasized or deemphasized (“HIM, I do not like” vs. “I HATE him”) through word ordering or intonation. As a consequence, it is unlikely that all information is equally weighted during processing. In the present study, we do not explicitly manipulate intonation or information structure, but we discuss sources of bias due to the unequal variation in semantic categories across part of speech and demonstrate a method that allows to infer rich representational content even in the presence of attentional biases.

### Measuring similarity through behavior

Perceived similarity can be measured by means of different behavioral tasks. Earlier used methods include asking people to freely sort a set of items into piles (free sorting, e.g., Bencini and Goldberg 2000), to make speeded same/different judgments (implicit measure, inter-item confusability, e.g., Sergent and Takane 1987), to determine the odd one out of three items (triad test, e.g., Roberson et al. (1999); Hebart et al. (2020)), to rate the similarity of two items on a scale (pairwise judgments, e.g., Migo et al. 2013) or to indicate similarity between items geometrically by placing them either close by when similar or far apart when dissimilar (Richie et al. 2020). In the current study, we implement a geometrical task because it provides several advantages over other methods. All of these methods can in principle be used to collect continuous similarity measures. Whenever binary similarity judgments are acquired, e.g., in free sorting or the triad test, continuous values can be obtained by combining data across multiple participants or repeated presentations of the same pair. Both pairwise judgments and geometrical tasks have the advantage of probing continuous valued similarity at the single participant level. Beyond that, geometrical tasks additionally allow for the most time-efficient sampling. This is because in a geometrical arrangement task, participants are asked to arrange several items, randomly scattered across a screen, within a circular 2D space, such that the distance between items is proportional to each pair’s similarity. Spatial adjustment (via drag and drop) of each individual item hence communicates multiple similarity judgments at once. The time to acquire pairwise similarity judgments, in contrast, grows quadratically as a function of total set size, since *n*(*n*-1)/2 judgments are necessary for a set of *n* items. In practice, the pairwise similarity judgements method has been shown to last 5–6 times longer as compared to a geometrical task on the same stimuli (Hout et al., 2013). Furthermore, the similarity ratings attained through geometrical tasks have been shown to correlate highly with pairwise similarity ratings. Therefore, geometrical tasks are to be preferred over other methods when sampling similarity judgments for larger sets of items as well as for more complex items such as sentences and complex scenes, which by their nature will require longer processing times than pictures of objects or single words.

**Fig. 1:**
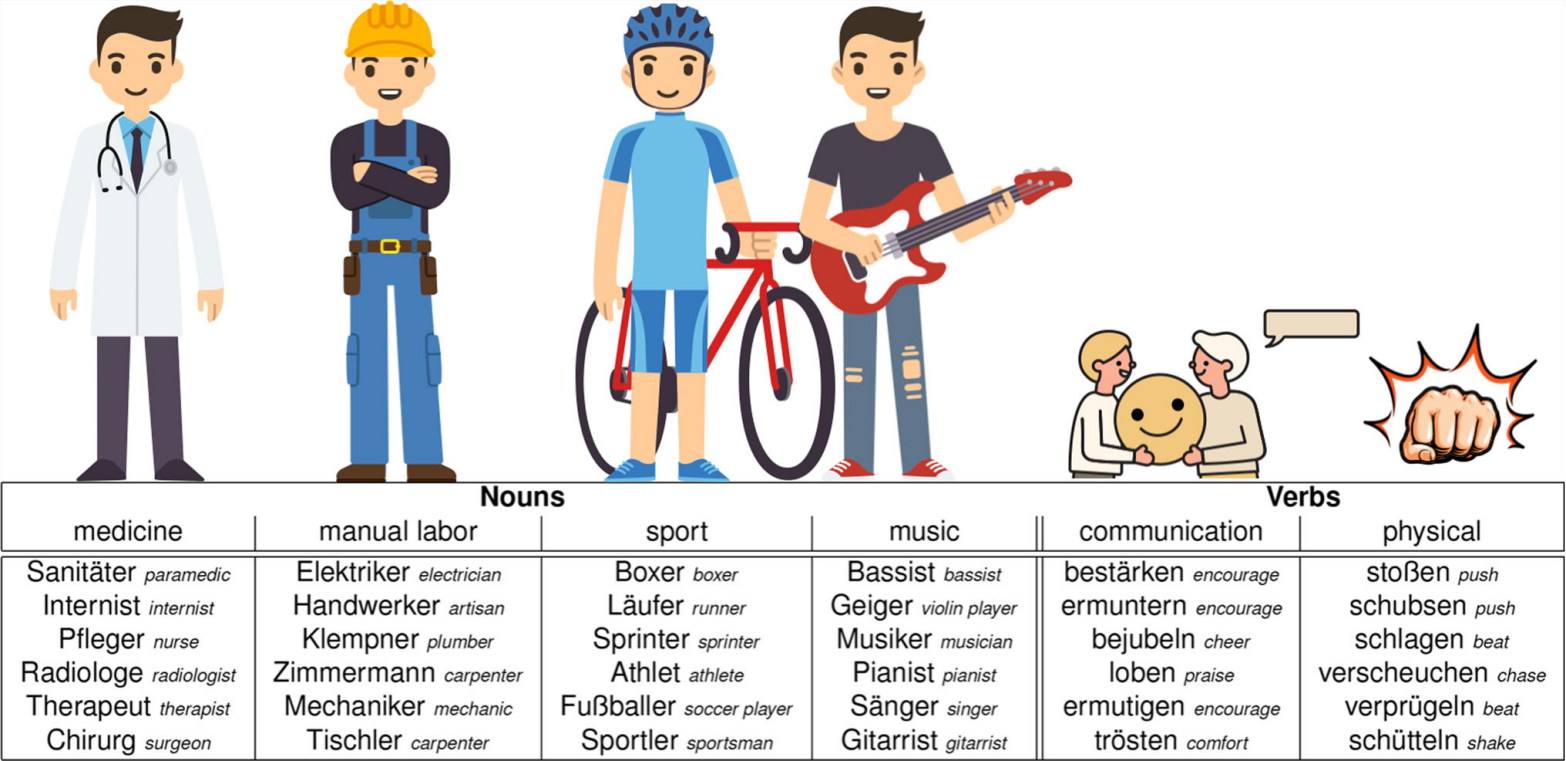
Stimulus vocabulary. Stimuli were presented in German as listed here. English translation in italic. Note that some words are homonyms in English but two distinct words in German

### High-dimensional representations

Pairwise similarity judgments across a set of items can be converted into a Cartesian space that illustrates the underlying representational configuration, for example through a technique called multidimensional scaling. Each item then is mapped onto a point in that space, such that the distance between items approximately corresponds to their relative similarity. The dimensionality of this Cartesian space depends on the number of dimensions along which two stimuli can be compared. This could be visual features such as color, shape, and texture in the case of visual stimuli or this could be semantic dimensions such as valence, topic, etc., in the case of words. Hence, the dimensionality of the approximated representational space does not depend on the task, but on the stimulus set and its perception by the subject. This means that despite their 2D nature, geometrical tasks can capture higher-dimensional representations already through a single arrangement of a set of items, as has been shown for both visual objects and single words (Richie et al., 2020; Hout et al., 2013). Recent extensions of the geometric arrangement task, which go beyond the single arrangement, have made it even more sensitive to high-dimensional representations by probing for similarity repeatedly and within different stimulus subsets, allowing for a more diverse and flexible selection of the relevant dimensions. Kriegeskorte and colleagues developed an extension of the geometric arrangement task, during which the subject sequentially arranges multiple displays, each containing subsets of the full item set (Kriegeskorte & Mur, 2012). The final representational dissimilarity matrix (RDM) is then computed by combining evidence across all subset arrangements. Past studies have successfully applied the multi-arrangement task to quantify high-dimensional mental representations of naturalistic images (King et al., 2019) and visual scenes (Groen et al., 2018), though none of them have modeled relational information specifically.

In the present study, we report online-acquired similarity judgments on (1) isolated nouns selected from four semantically distinct profession categories and on (2) transitive sentences containing those nouns. The sentences described events of one person acting on another (e.g., “Today the surgeon comforted a carpenter”). Verbs across sentences were chosen from two distinct semantic categories. Any pair of sentences differed in whether they contained a verb and or nouns from the same versus different semantic categories. Moreover, sentences differed on the relational information they encoded about agent and patient roles of shared nouns. We expected that perceived similarity of isolated nouns would be fully determined by their semantic category. The results of the noun task serve as a baseline for the sentence task, verifying that the selected stimulus material evokes the assumed word semantics and can be used to assess the influence of thematic role assignment on similarity in sentence contexts. Based on similarity judgements on the full sentences, we then evaluate the underlying dimensionality of integrated sentence meaning. We expect that beyond the semantic categories of the nouns alone, their assignment to thematic roles as well as the semantic category of the verb will further modulate similarity judgments. We demonstrate that factorization can be used to recover relational information such as thematic role assignment in the presence of strong attentional biases towards the verb. Furthermore, we suggest an additional use case of sentence similarity judgments, namely, as a point of comparison for computational models of human sentence processing.

## Methods

### Stimuli creation

We created a set of sentences (*n* = 48) describing simple transitive events, such that the similarity between events could be captured by a small number of meaning dimensions. For this, we selected 36 words that belonged to six semantic categories, i.e., 24 nouns & 12 verbs describing four professional domains (medicine, manual labor, sport, and music) and two action domains (communication and physical interaction) respectively (see Fig. 1). Stimuli were created in German. Across semantic categories, words were matched (nouns and verbs separately) according to number of letters, number of syllables, and frequency. In addition, we took care that noun categories did not systematically differ from each other in their suffixes. Importantly, we chose words that could be combined more or less arbitrarily without imposing strong constraints with respect to meaning amongst each other. For example, a surgeon can engage in either physical (e.g., pushing someone) or communicative (e.g., praising someone) actions as both the agent and the patient of the event.

**Fig. 2:**
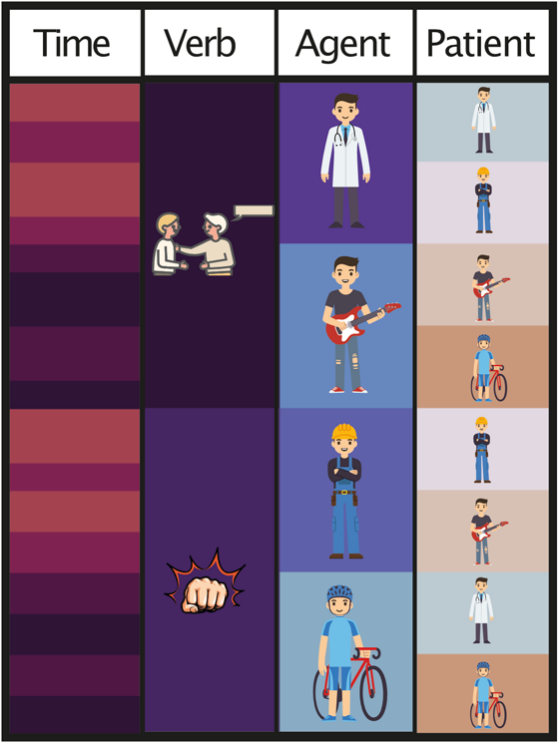
Stimulus randomization. The diagram illustrates for all sentences (rows) which elements make up any given sentence. Elements are color-coded to indicate the identity of the temporal adverbs (1st column), the semantic category of the verb (2nd column), the semantic category of the agent role (3rd column) and the semantic category of the patient role (4th column). Given any sequence of adverb, verb, and agent, the patient semantic category is uncertain, since there are always two categories that occur with equal probabilities

From this vocabulary of 36 words, we formed sentences by pseudorandomly combining nouns and verbs (e.g., “This morning the paramedic praised the electrician.”, see full stimulus set in Appendix). The randomization was generated according to six constraints: Nouns were combined such that each noun in agent position would be (1) paired equally often with a patient from either the same semantic category or one of the other semantic categories (e.g., “the paramedic praised the electrician.” & “the paramedic encouraged the nurse.”). Also, each noun (2) appeared in both object and subject position (e.g., “the paramedic praised the electrician” & “the boxer hit the paramedic”) and (3) each noun category appeared equally often. Subsequently, noun pairs and verbs were combined such that (5) each noun category would occur equally often with verbs from both action categories. Finally, in the beginning of each sentence, we added a temporal adverb (e.g., “This morning”,“Yesterday” etc.) and then constructed the sentence according to VSO word order. The temporal adverbs were distributed such that (6) each adverb could precede two of the four noun categories and either of the verb categories (see Fig. 2).

We formalize our predictions about the expected pairwise sentence similarity in two reference measures. The first reference quantifies sentence similarity based on bag-of-words sentence representation by counting how many noun and verb semantic categories were shared across two sentences. The second reference, hereafter referred to as “relational reference”, quantifies sentence similarity based on not only the semantic categories but also the thematic role assignment. We count how many nouns from the same semantic category occur in the same thematic roles across two sentences and whether or not the verbs share the same semantic category. As a result, values of predicted similarity range from zero to three. For example, sentences with different semantic categories for their nouns and verbs are expected to have zero similarity under both references. However, when the nouns are of the same semantic category but in reversed thematic roles, the relational reference measure gives a score of zero while the bag-of-words measure gives a score of 2 (see Table 1 for further examples). Our definition of relational predictions in this paper is minimally constrained and does not take into account any possible hierarchical effects of thematic roles on comprehension. This allows for equal contribution from both agent and patient roles towards the expected similarity score. However, our method can also be applied to more complex models of thematic role representations, such as those suggesting a hierarchy of thematic role assignments (Baker, 1996; Rissman et al., 2015). If this were the case, our method might result in a lower fit with our relational reference measure because in our measure all thematic roles are equally weighted. Nevertheless, since there is little agreement on thematic hierarchies and most theories assume a general prominence of both agent and patient over other roles (Rissman & Majid, 2019), we decided to predict similar effects for both roles. Finally, we had no prior theoretical expectations about the similarity structure of temporal adverbs. Nonetheless, our analysis recovered patterns of perceived similarity elicited by the adverbs as we will demonstrate.

**Table 1:** Example sentences

1	Today the surgeon comforted a carpenter
	* Heute tröstete der Chirurg einen Tischler*
2	Earlier, the nurse encouraged the plumber
	* Vorhin bestärkte der Pfleger den Klempner.*
3	Today the mechanic beat up a plumber
	* Heute verprügelte der Mechaniker einen Klempner.*
4	In the morning a sprinter pushed the athlete
	* Am Vormittag schubste ein Sprinter den Athleten.*

Sentences are listed in English translation (original German stimuli in italics below). 1 and 2 are considered most similar because semantic categories of agent, patient and verb are shared. 2 and 3 are more dissimilar in comparison, since verbs and agent are of different semantic categories. Note that sentence 2 and 3 might have a higher visual similarity due to sharing the identical final noun, but our predictions are on the semantic and relational similarity of the sentences Sentences 1 and 4 are most dissimilar since they do not share any semantic category

**Fig. 3:**
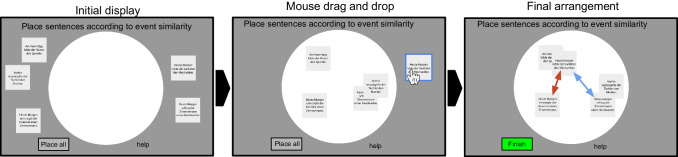
Illustration of computer-based task. In the initial display, sentences are arranged randomly outside the arena. In order to continue to the next trial, all sentences need to be placed per drag-and-drop inside the white arena. Sentences are selected by clicking on them and can then be moved around. Items can be placed and replaced multiple times. Items can be stacked on top of each other. Once all items are inside the white arena, the Finish button on the bottom will become active and upon clicking on the button the next trial will be initiated. Distance between items will be measured from the center of the square containing the sentence

### Online multi-arrangement task

Two hundred native German speakers rated the perceived similarity of our stimuli. During the multiple arrangement task, participants were asked to arrange the nouns or sentences on a computer screen inside a white circular arena by using computer mouse drag and drop operations (see Fig. 3 for illustration). The distance of the placed sentences indicates the perceived similarity. Usually, participants would see the full stimulus set on the first trial and subsequent trials consist of a subset of those stimuli. For the subset selection, we rely on an adaptive procedure developed by Kriegeskorte and Mur aimed at optimizing the trial efficiency given the evidence and utility of each item pair. The evidence for a given item pair similarity is computed as the square of the signal-to-noise ratio for that pair. The signal-to-noise ratio is proportional to the actual on-screen distance. This is possible because although distances are relative and have meaning only in the context of a single trial, the distortion of each drag-and-drop placement due to a small placement error is assumed to be constant across trials. If the distance between two items is small to begin with, then the placement error will lead to relatively stronger distortion, and hence lower signal-to-noise ratio.

The utility of presenting any given item pair is defined as a saturating function $u(w) = 1-e^{-w*d}$ of the current evidence *w* of the same pair. This function ensures that additional evidence is weighted higher when there is only little evidence for the similarity judgement and vice versa. In our study, we set the exponent d = 10, such that the utility is saturated when evidence reaches 0.5, which is the default value proposed in the original implementation by Kriegeskorte and Mur. Since we could not present all items on the first display the evidence for not yet seen item pairs will be zero after the first trial and those items will be sampled until there is some similarity judgement logged for all item pairs. Subsequently, the item pair with the least evidence will be selected for the next subset. New items will be repeatedly added to this subset, choosing items that maximize trial efficiency first and halting as soon as any additional item will reduce trial efficiency. A more detailed description of the subset selection procedure can be found in the publication introducing the multiple arrangement method by Kriegeskorte and Mur (Kriegeskorte & Mur, 2012). A consequence of the selection procedure is that on later trials, displays contain fewer items. This allowed participants to refine their judgments with distinctions that are more difficult to express in the context of the whole set and the limited arena space. To extract one single estimate of all pairwise similarities, the overlapping subsets are first scaled and then combined as weighted averages (see Kriegeskorte and Mur (2012) for details). Due to the iterative procedure, the task is very efficient at obtaining reliable high-dimensional similarity judgments for our 48 individual sentences within 60 min per participant (or 24 nouns within 30 min). The behavioral data were collected using the Meadows web-based platform for psychophysical experiments (http://meadows-research.com). Online participants were recruited from the Prolific online participant pool (http://www.prolific.ac.uk). All subjects gave informed consent via button click before participation and received monetary compensation according to Prolific guidelines.

Half of the participants were presented with the nouns that were used to generate sentences and the other half rated the full sentences. Subjects were instructed to place all nouns/sentences inside the white area in a manner that reflects the similarity between the described people/events. The instructions did not specifically mention that people could be categorized into professions or that verbs could be categorized into positive and negative actions. Nonetheless, the majority of subjects mentioned those dimensions in their debrief. In addition, those subjects seeing the events were instructed to not place items only according to a single word in the sentence but rather pay attention to all words. Prior to the main task, an example was shown for how an arrangement could look, using nouns/sentences that were not part of the stimulus set.

#### Noun similarity

For the noun similarity task, we collected arrangement data from 100 subjects (mean age = 31, SD = 9), of which 41 were female. Subjects arranged all 24 unique nouns on the first trial and each subsequent trial contained subsets of those nouns. Subsets consisted of a minimum of three and a maximum of 24 nouns. There were no unseen item pairs and we did not exclude any of the subjects. On average, subjects completed 37 trials (SD = 8) before they either reached a minimum evidence level of 0.5 or 30 min had passed.

#### Event similarity

To our knowledge, we are the first to apply this task to full sentences instead of individual words or pictures. In order to present the sentence stimuli in a format suitable for the task, we split each sentence into 3–4 lines, such that it could be presented within a square box. In order to minimize the influence of the verb, sentences were broken up, such that the verb never appeared on a line on its own. In the sentence similarity judgment task, an additional practice trial preceded the main task. The practice was based on three sentences, of which two were semantically synonymous and the third describing a completely different event. The main task was complete once a subject had reached a minimum evidence level of 0.5 for each item pair or 60 min had passed. On average, subjects completed 112 trials (SD = 41). Due to space limitations, participants were not presented with all sentences in the beginning. Instead, they saw only ten sentences during the first trial, and at least three items or up to a maximum of ten on each subsequent trial. Fourteen subjects were excluded because they executed too few trials within the 60 min, i.e., they rated less than 50 item pairings. The remaining 86 subjects (mean age = 30, SD = 9; 38 female) were all German native speakers.

### Matrix factorization

We used non-negative matrix factorization (NMF) (Lee & Seung, 2000) to investigate which underlying dimensions played a role in the sentence similarity ratings. For this, we first concatenate all individual subject RDMs into a large Matrix D of dimensions number of subjects $\times$ number of item pairs. NMF allows us to decompose D into two non-negative matrices, which gives a lower rank approximation for D. Basically, we decompose this matrix into two matrices, such that $D = W \times H$, where W is the $|n| \times k$ mixing matrix that contains the weights for constructing N observed subject similarity judgments from the k components, and H is a $k \times |t|$ factorization matrix that contains the k latent components capturing underlying pattern of pairwise item similarity. Note that, by definition, all $H_{i,j} >= 0$. We applied the NMF implementation of scikit-learn (Pedregosa et al., 2011), which finds the optimal decomposition by iteratively optimizing the distance between D and the matrix product WH using the squared Frobenius norm as the distance function.

The NMF algorithm requires to specify the number of latent components k to be extracted. In order to get an estimate of what the optimal k would be, we computed the NMF repeatedly (*n* = 1000) with different random initializations, each time limiting the factorization to an increasing number of components (1 < k < 20). For each of the 1000 random initializations, we checked for each additional component for how many subjects it would receive maximal mixing weights. Although each additional component will further optimize the fit to the data, we only regard it as informative, if it captures general judgment patterns, i.e., receive maximal weights for multiple subjects, rather than individual solutions. On average, it took seven components to capture all patterns in the data, which generalized across at least two subjects. We then fixed the number of components to seven and again computed 1000 factorizations using different random initializations. Based on the resulting 7000 components, we ran agglomerative hierarchical cluster analysis to determine which underlying components are reliably found throughout repeated factorizations. Based on visual inspection of the within- and between-cluster similarity, we decided on a distance threshold of 0.85, such that we could define five clusters of components, which would reliably emerge across multiple factorizations (at least 720 times out of 1000) and were for the most part interpretable in terms of the underlying similarity patterns (see Fig. 5). From each cluster, we computed one final component, by taking the average across all cluster exemplars (centroid). Based on the resulting five components, we again computed the unmixing matrix using the same optimization algorithm. In order to qualitatively assess the final factorization, we computed the Spearman rank-ordered correlation between each of the components and the upper triangular vectors of our binary model matrices for the verb category, the agent category and the patient category, respectively.

**Fig. 4:**
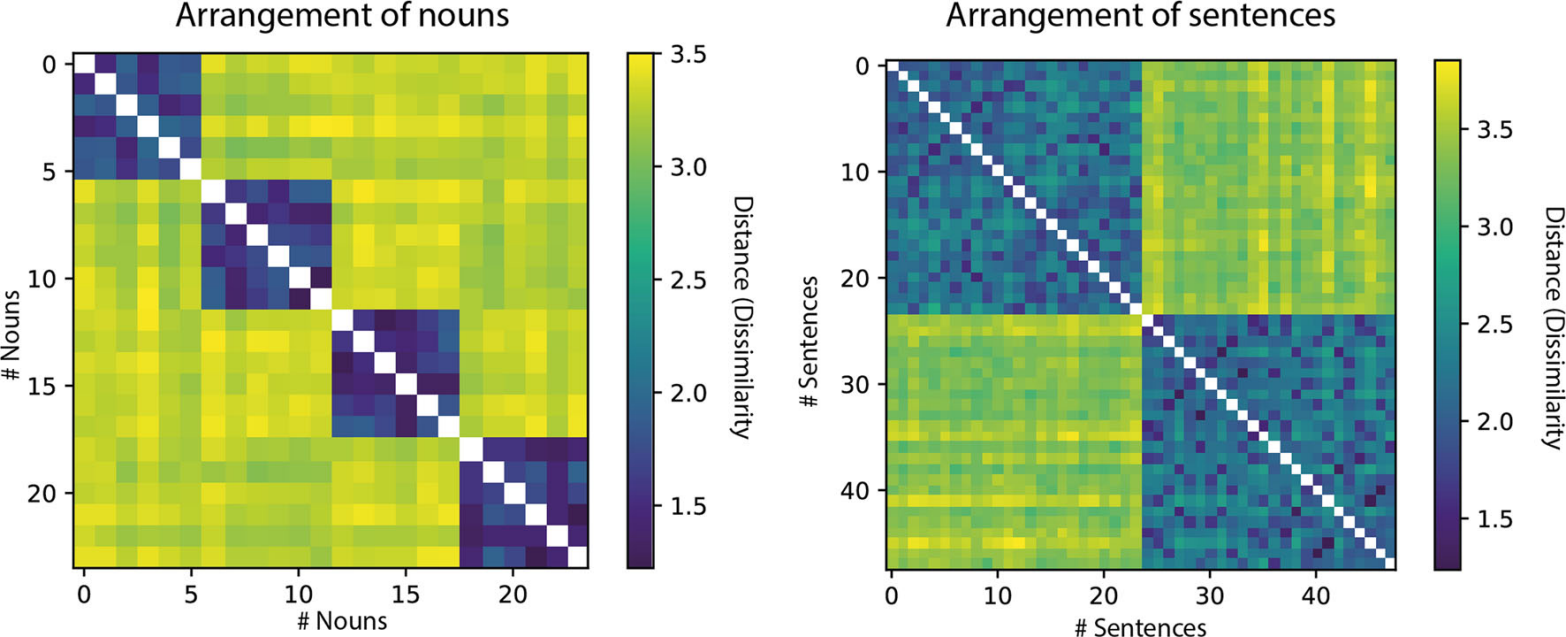
Mean dissimilarity for nouns and sentences. *Left* Nouns are sorted (along rows and columns) according to thematic categories (order: medicine, manual labor, sports and music) and within each category the same order as depicted in Fig. 1 is maintained. *Right* Sentences are sorted according to the full stimulus list (see appendix or Fig. 2), i.e., all sentences containing communicative verbs and both agent and patient from the category “medicine” first, followed by all sentences containing communicative verbs and agent from category “medicine” plus patient from the category “manual labor” etc

**Fig. 5:**
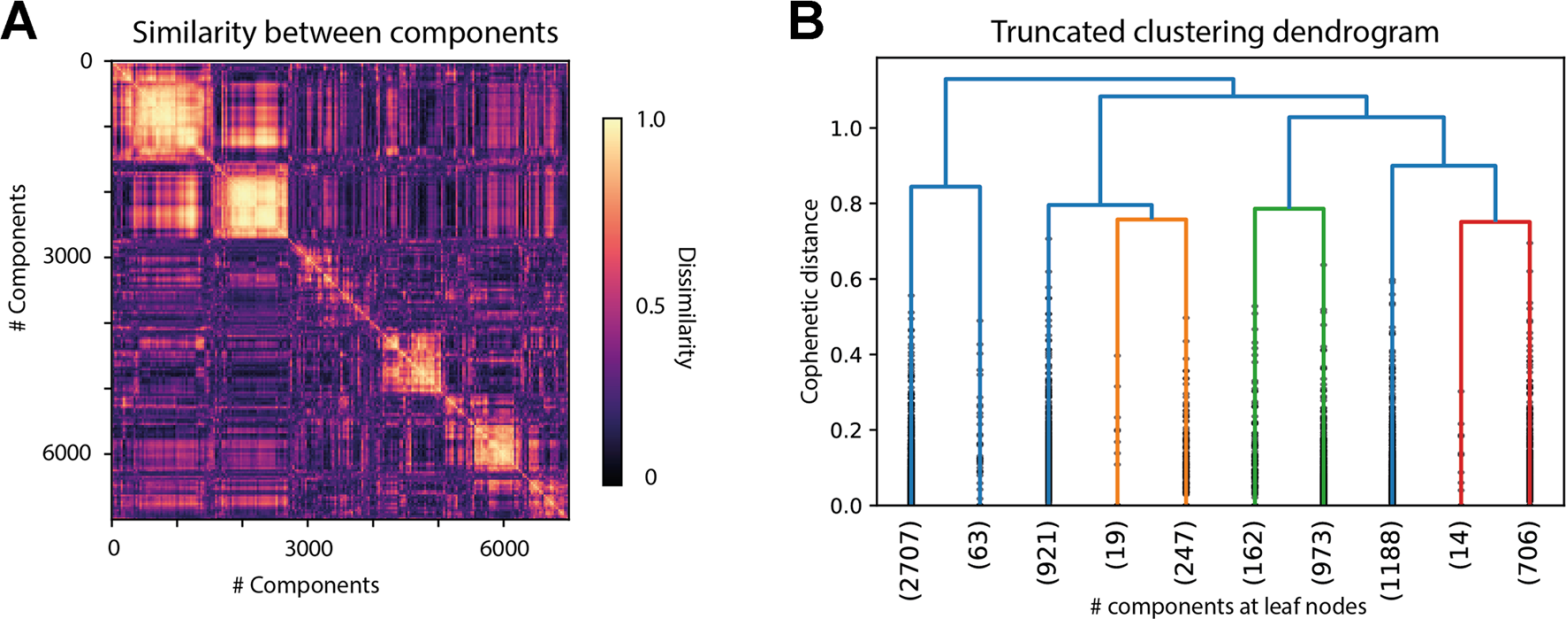
Clustering of latent factors driving event similarity task results. **A** Pairwise similarity between all components (1000 repetitions of factorization into seven components each yields 7000 components in total) is depicted. Lighter color codes for similarity (absolute Pearson correlation). Components are sorted according to order determined by hierarchical clustering algorithm. **B** Truncated dendrogram showing only the last ten merges across all components. On the *x*-axis, for each cluster the label indicates the amount of leaf nodes (components) belonging to the depicted cluster. *Horizontal lines* indicate a merge of leaves into a new cluster. The height of the horizontal lines indicates the distance between the merged sub-clusters. As can be seen, some sub-clusters merge only relatively few components (e.g., 19 in left branch of orange cluster). The threshold of distance 0.85 for determining clusters was in part motivated to result in roughly equally sized clusters (**B**) that seem most coherent based on their inter- & intra-cluster similarity (**A**)

### Sentence similarity based on ANN generated embeddings

For our sentence stimuli, we extracted sentence embeddings from three pre-trained ANN models, GPT2 (Radford et al., 2019), BERT (Devlin et al., 2018) and SBERT (Reimers & Gurevych, 2020b), and compared their pairwise similarity to our behavioral similarity judgments. For the BERT and GPT2 architectures, embeddings were extracted from models trained on German texts and implemented in PyTorch with the Huggingface module. Specifically, we used the bert-base-german-cased model (https://huggingface.co/bert-base-german-cased) and german-gpt2 https://huggingface.co/dbmdz/german-gpt2). For BERT embeddings, we extracted activation based on units from layer 12 and special token “[SEP]”, which marks the end of a sentence. For the GPT2 embeddings, we extracted activation based on units from layer 12 and the final word token, ignoring punctuation. For the SBERT architecture, we used a pre-trained model architecture implemented in PyTorch with the Sentence-transformers module https://www.sbert.net/. SBERT is not available in a German-only version, so we used the multilingual model distiluse-base-multilingual-cased, which supports a range of languages including German (Reimers & Gurevych, 2020a).

## Results

The multi-arrangement task provides pairwise distances (dissimilarities) for all item pairs (e.g., pairs of nouns or pairs of sentences). These pairwise distances for *n* items can be visualized as a so-called representational dissimilarity matrix (RDM) $H = n \times n$, such that each entry in the matrix $H_{i,j}$ contains the dissimilarity between item *i* and item *j*. For both groups of subjects separately, we extracted one continuous matrix by first normalizing the individual subject RDMs by their standard deviation, and then averaging over all subjects. Figure 4 depicts the resulting group averages.

**Fig. 6:**
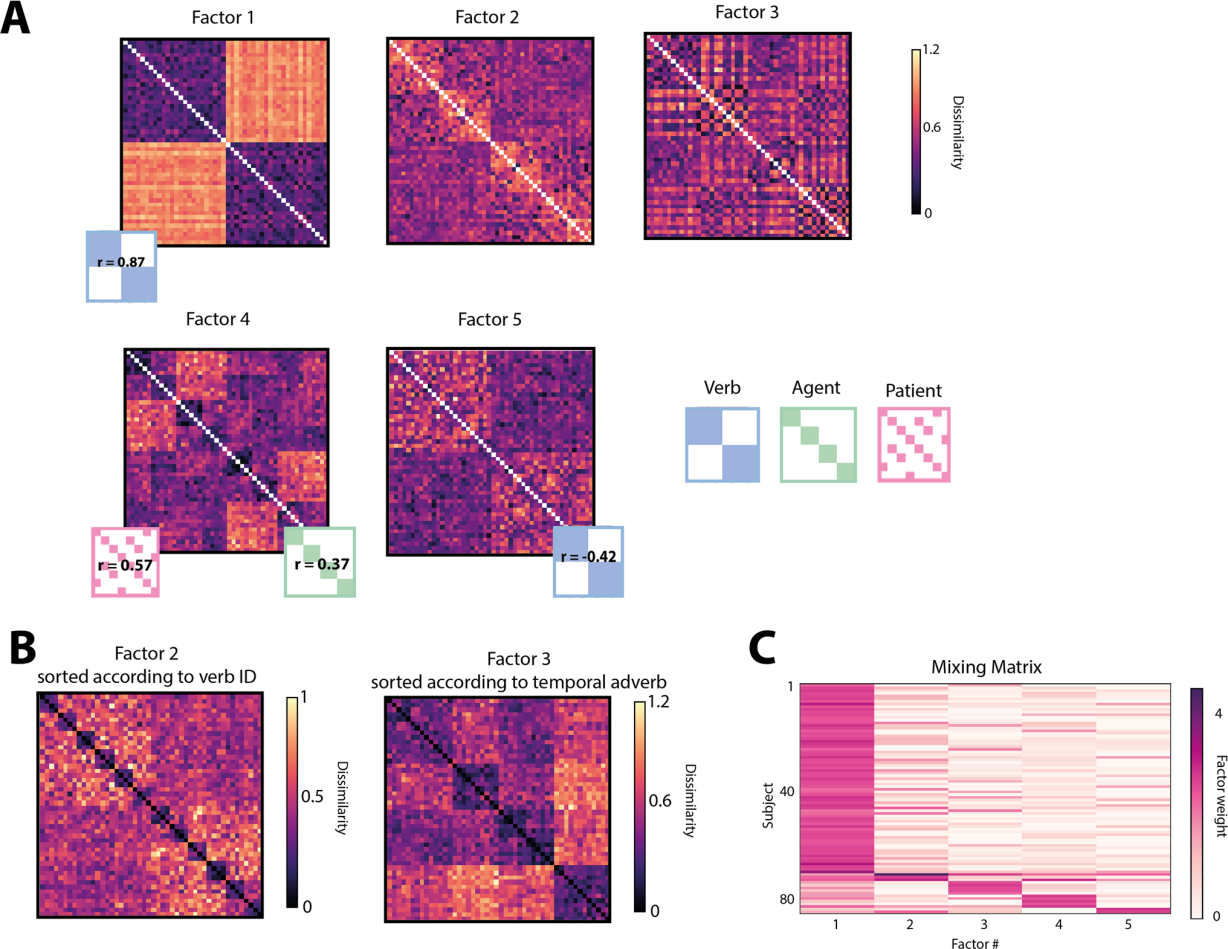
Latent non-negative factors. **A** Each factor is visualized as a sentence-by-sentence matrix (*n* = 48) with sentences in the same order as listed in the full stimulus list (see appendix or Fig. 2), i.e., all sentences containing communicative verbs and both agent and patient from the category “medicine” first, followed by all sentences containing communicative verbs and agent from category “medicine” plus patient from the category “manual labor” and so on and so forth. For each factor, its Spearman correlation with the theoretical semantic category models for verb (*blue*), agent role (*green*) and patient role (*pink*) is shown for those semantic dimensions best capturing the similarity pattern expressed by the factor. Both factors 2 and 3 were poorly correlated with any of the semantic category models. Their patterns are visualized by re-ordering sentences according to verb ID (**B**, left) and temporal adverb identity (**B**, right), respectively. The order of the temporal adverbs was the following: “Today” / “This morning”, “Earlier”,“In the morning” or “Yesterday” / “Yesterday evening”. **C** Mixing weights are depicted per subject (*rows*) and factor (*columns*).* Darker color* indicates higher weights. Subjects are sorted according to their maximal factor weight

Results for the noun similarity task reflect that subjects easily picked up on the semantic categories and arranged nouns according to profession. The average dissimilarity within any given category was lower (mean = 1.6, SD = 0.08) as compared to the average dissimilarity across categories (mean = 3.3, SD = 0.04) and the correlation with a binary, descriptive model of noun similarity, encoding all within-category pairs with a distance of 0 and all across-category pairs with a distance of 1, was high (rho = 0.71). In the event similarity task, the average dissimilarities are most highly correlated with a binary descriptive model of verb category (rho = 0.86). Based on the average, it is therefore not clear whether participants arranged sentences only based on the verb categories (i.e., positive communication actions versus negative physical actions) or whether they additionally took into account the thematic role assignment of noun categories (professional domains) to agent and patient roles (Fig. 5).

**Fig. 7:**
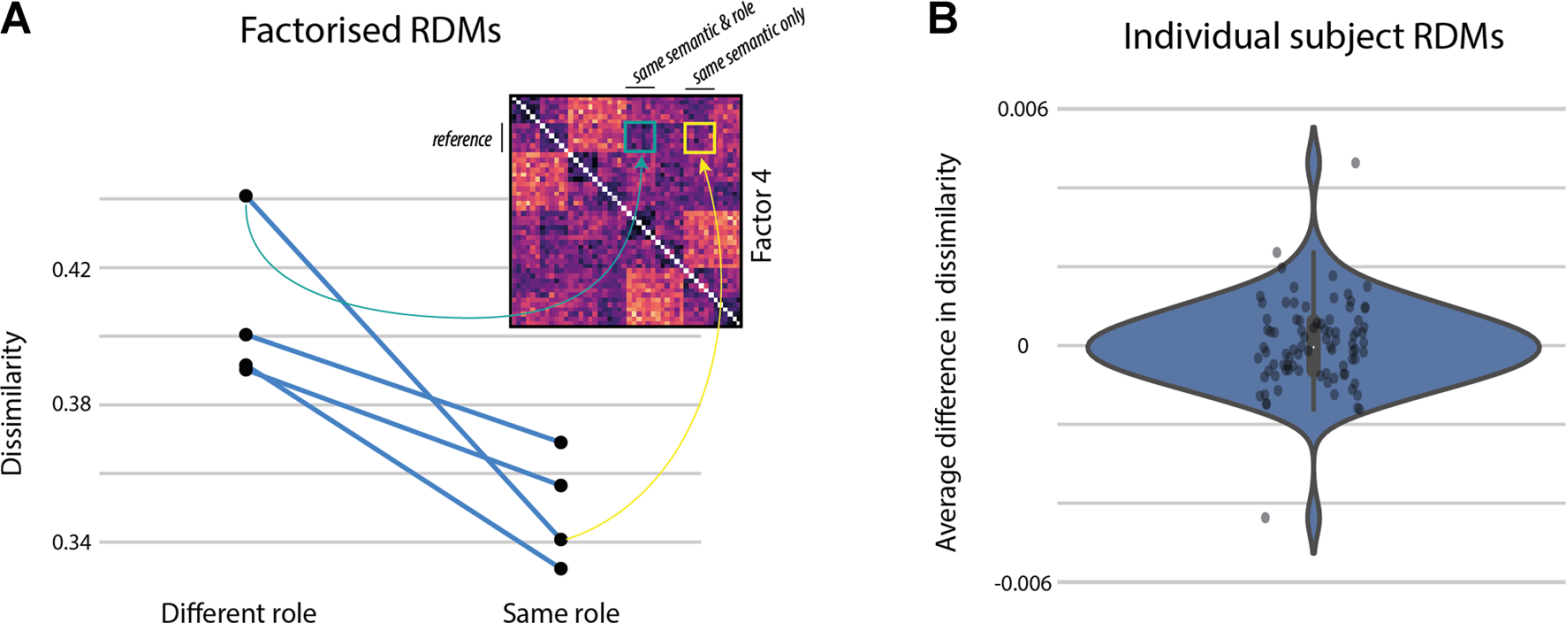
Cross-sentence correlations controlled for semantic overlap. Average pairwise dissimilarity is shown for two subsets of sentences, that either share a noun semantic category in the same or a different thematic role with a third subset of sentences (the reference). **A** Dissimilarities are shown based on component four after factorizing the RDMs. Each point plots the average dissimilarity across one distinct subset of cross-sentence dissimilarities. Pairs of subsets within which semantic overlap is controlled are connected through lines. **B** Difference in dissimilarity is shown for each individual subject. For each pair of subsets, the difference in dissimilarity was computed. Each point plots the average difference in dissimilarity across all four subsets per participant

### Factorizing high-dimensional representations

Even though it seems as if event similarity ratings are mostly driven by the semantic category of the verb, subjects reported multiple strategies for solving the task. Indeed, a data-driven factorization revealed underlying components that influenced similarity judgments beyond verb semantics. We identified five components (see Fig. 6A), which robustly resulted across 1000 factorizations with random initial weights and together explain more than 95% of variance in the data. The first component reflected the verb similarity (correlation with a binary verb category model was 0.87). The second component did not reflect any of our categorical dimensions, instead, after inspection we found that it reflected sorting according to verb identity, i.e., whether two sentences shared the same verb or not (see Fig. 6B left). Component three reflected sorting according to the temporal adverb of each sentence (see Fig. 6B, right). Finally, component four reflected sorting of sentences according to semantic similarity of both the agent (rho = 0.57) and the patient (rho = 0.37) of the sentence. Component five remained elusive but was negatively correlated with the model for verb semantics (rho = – 0.42). Based on the mixing matrix (see Fig. 6C), we observed that similarity according to verb semantic category was weighted highest for most subjects. Nonetheless, the majority of subjects took into account additional semantic dimensions when arranging the sentences, namely the specific verb identity, the temporal information, the semantics of the agent role, and the semantics of the patient role.

The correlation with the agent and patient category models in component four provides a first indication that relational information is influencing peoples’ similarity reports to some degree, the patient role of a sentence being more influential compared to the agent. Importantly, within each model, both semantic and structural information is taken into account. For example, the agent and patient model RDMs code sentences as highly similar if they share a noun from the same semantic category in the same relational role (e.g., as agent or patient, respectively) and as dissimilar if they either share a noun in a different role or if they don’t share the same semantic categories at all. Therefore, it could be that model correlations are partly driven by the mismatch in semantics across sentences and not just the mismatch in relational information.

**Fig. 8:**
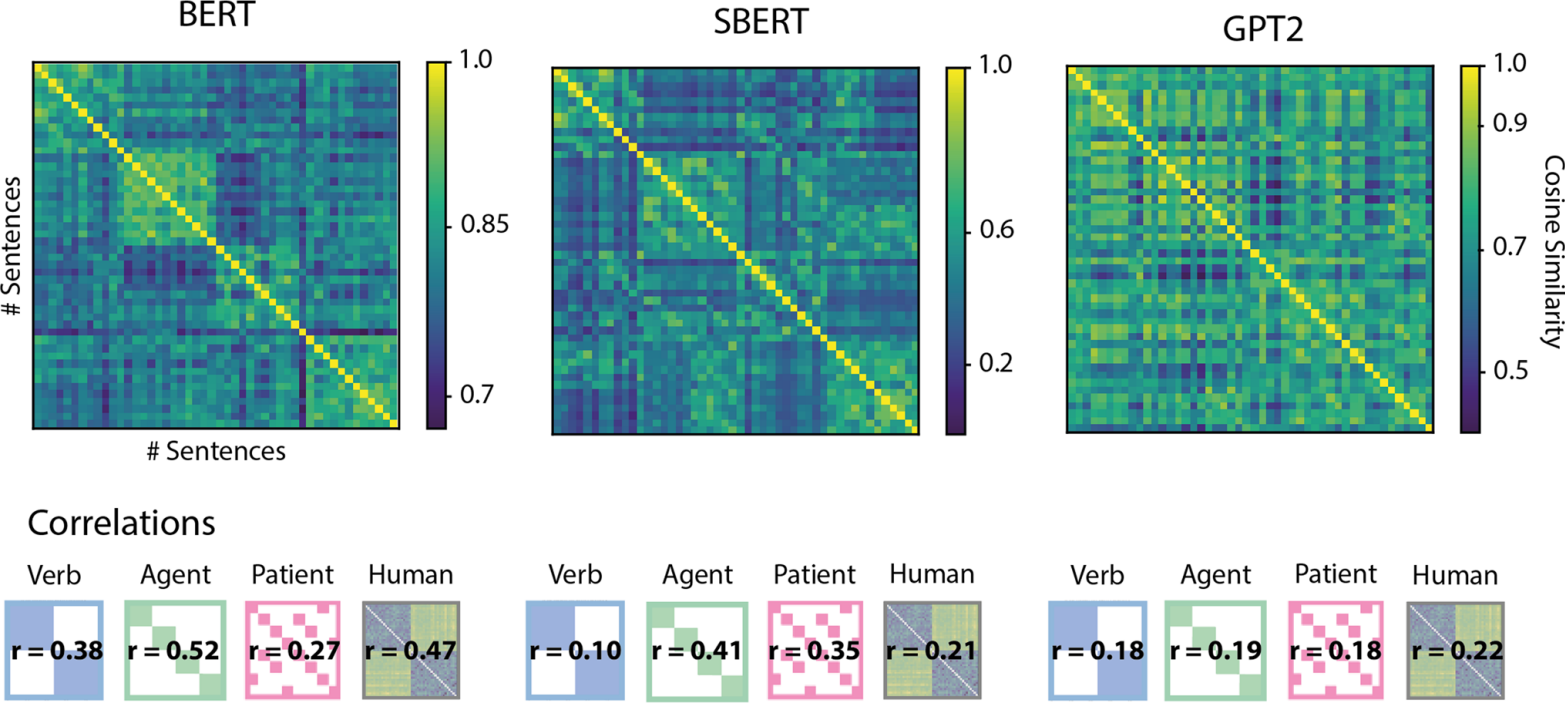
Representational similarity matrix for all stimulus sentences based on ANN generated embeddings. Pairwise cosine similarity is plotted for each sentence pair $n_{i,j}$ (*i* = 1,2,...,48 and *j* = 1,2,...,48) of the stimulus set and three different ANN architectures. Sentences are sorted according to the full stimulus list (see appendix or Fig. 2), i.e., all sentences containing communicative verbs and both agent and patient from the category “medicine” first, followed by all sentences containing communicative verbs and agent from category “medicine” plus patient from the category “manual labor” and so on and so forth. Pearson correlation coefficients for theoretical models of either verb (*blue*), agent (*green*), patient (*pink*) semantic category as well as correlation with human data are depicted below each model

To test whether relational role information modulated similarity ratings, we devised a comparison between subsets of sentences, that controls for semantic overlap. This was achieved by comparing the average pairwise similarities across subsets of sentences, where three given subsets were selected such that they share the semantics of one noun, but only two of the subsets additionally share the thematic role it appeared in. For example, we averaged over all pairwise similarities between events describing medical professionals acting on manual labor professionals and either (1) events describing manual labor professionals acting on each other (shared semantics & role, teal square in Fig. 7A) or (2) events describing athletes acting on medical professionals (shared semantics only, yellow square in Fig. 7A). If thematic roles influence similarity judgments, those sentences sharing both semantic category as well as corresponding role should be judged as more similar compared to when they share semantics only. We computed this difference in perceived similarity on all possible subsets, controlling for semantic overlap and verb semantics within each comparison. Further, we computed this comparison based on both the factorized results, specifically component four as well as on all subject-specific RDMs. When computing this contrast on each subject’s original RDM, the average difference in perceived similarity over all subjects was not significantly different from 0 (*p* = 0.46, one-sided *t* test, (Fig. 7B)). The same analysis run on the factorized data, however, revealed clearly that similarity was higher between subsets of sentences sharing both semantics and relational role compared to those sharing only semantics (mean difference = 0.06 normalized dissimilarity, *p* = 0.039 one-sided *t* test on mean subset similarity). This was true for all four different pairings of subsets (Fig. 7A). Hence, once the verb bias is factored out, the results reveal an effect of relational role information on similarity judgments.

### Sentence similarity based on ANNs

We evaluated the pairwise similarity based on sentence embeddings generated by three ANN models. All models produced embeddings that captured verb, agent, and patient categories to some extent (pairwise cosine similarity between sentence pairs was on average higher for items that shared categories than items with different categories across all three dimensions). Nonetheless, we observed differences in the strength with which each model captured different dimensions of event meaning. For example, while the GPT2 model had overall a low fit to our relational reference, it captured each dimension more or less equally. In contrast, both BERT and SBERT produced embeddings that loaded more strongly on certain dimensions. While BERT embeddings most strongly encoded the agent and the verb dimensions, the embeddings produced by SBERT contain less information about verb semantics. SBERT in turn appears to better encode the patient role filler compared to the other models. Interestingly, while SBERT is the only model optimized specifically for pairwise sentence similarity judgments (the training goal most resembling the multiple arrangement task), it is also producing sentence embeddings with the worst fit to our observed human behavioral data (see Fig. 8). Out of all three models, BERT produces similarities that are most correlated with the human judgments. While sentence similarities according to GPT2 in comparison have lower correlations with human data, they match the human data better than any of the individual meaning dimensions.

## Discussion

We applied a geometrical multiple arrangement task to acquire similarity judgments for 24 nouns and 48 sentences describing simple transitive events. Similarity judgments revealed a sensitivity to the semantic category of professions when arranging nouns. Although sentences contained those same nouns, the semantic category of profession was less prominent in the sentence-by-sentence similarity judgments. Instead, average sentence similarity judgments seemed to be highly sensitive to the semantic category of the main verb. Matrix factorization, however, revealed that subjects additionally took into account both the semantic domain of the nouns as well as relational role information when arranging the sentences.

We observed that the similarity judgments are more correlated with a model of the noun semantic category model of the patient noun compared to the agent noun. This demonstrates that the thematic roles do not affect sentence comprehension equally. The direction of the difference contradicts previous eye-tracking results (Wilson et al., 2011), which suggested a prominence of the agent role. Our data, in contrast, point towards a more prominent effect of the patient role on offline similarity judgments. Interpretation of this result should be cautious, however, because we did not vary sentence structure. Therefore the order of role is confounded with order of words.

Overall, the multi-arrangement task is suited for efficient sampling of similarity judgments even for linguistic stimuli beyond the single-word level. Importantly, combined with matrix factorization, it allowed us to quantify complex mental representations of integrated meaning.

### Verb bias

#### event templates

We found the verb similarity to be the dominant dimension according to which subjects arranged the sentences in our study. Although our study design does not directly test what causes the prominence of the verb, we can speculate about possible explanations for this finding: First of all, the importance of the verb may not come as a surprise given its special linguistic status in the sentence. It has been argued in the past that subtle features of verb semantics, such as subcategorization information, immediately affect online comprehension and can even be exploited to predict sentence structure (Hare et al., 2004; McRae et al., 1997). Furthermore, verbs are thought to be linked to so-called “event templates” (Tenny, 1994; Jackendoff, 1992; McKoon & Macfarland, 2002). Event templates formally conceptualize an event by establishing which primitive “event kind” it belongs to and by specifying the syntactic argument positions of its entities in a sentence. In our stimulus material, the physical verbs can be said to instantiate the semantic primitive ACT(x,y). This means that the event involves entity x acting upon entity y, where x and y map onto syntactic subject and syntactic object, respectively. The exact meaning of the verbs will further specify the event, e.g., entity x is acting upon entity y through negative, physical impact for a verb like “to hit”. The verb “to break”, on the other hand, would instantiate a very different event template, i.e., CAUSE(a,BECOME in STATE(x)), where entity x undergoes a change of state (intact to broken) through external force of a. The two verbs “to break” and “to hit” hence differ in the number of sub-events that it takes to characterize them. It has been experimentally demonstrated that such event templates are implicitly taken into account as we process sentences. For example, words that are presented in the same template across sentences can prime each other later on McKoon and Ratcliff (2008) and more complex event templates will slow down reaction times during lexical decisions (McKoon & Macfarland, 2002). In the present study, people may have been naturally biased towards attending the verb of a sentence (rather than the nouns) given that it carries crucial information about the event template.

#### Interactions between verb and noun semantics

Verbs can not only determine an underlying event template but they also directly shape our representations of event participants. This is because relational features of nouns interact with verb semantics in complex ways. For example, if sentences contain bidirectional verbs (e.g., John greeted Mary vs. Mary greeted John), their perceived similarity under role reversal might decrease less as opposed to sentences describing more unidirectional actions, since the role assignment of agent and patient becomes ambiguous. Furthermore, roles are not rigidly defined in terms of syntactic arguments only (e.g., subject, object) but carry semantic content (Holyoak, 2005). For verbs that convey a mental state, such as “to surprise” or “to notice”, the roles of the agent and patient can be defined as the causal element of the experience (the stimulus) and the undergoer of the experience (the experiencer), respectively. There exists an asymmetry between Subject–Experiencer and Object–Experiencer thematic structures. Specifically, the mapping between syntactic subject and syntactic object on the one hand and agent/stimulus and patient/experiencer roles on the other will depend on the specific verb semantics. For example, the cat (subject) surprising in example 1 below, maps better onto the woman (object) being noticed than the deer (subject) noticing in example 2 since they are both causal for the events described (Frankland & Greene, 2020b).

1. The cat surprised the man.
2. The deer noticed the woman.

In the current study, verb–noun combinations were chosen to be somewhat arbitrary to allow for role-reversal given the same verb (e.g., “the electrician encourages the guitarist”, “the guitarist pushes the athlete”). Nonetheless, verbs were unidirectional and will likely have modulated the semantic feature space evoked by a given thematic role. The agent of “to beat” might be perceived as an aggressive person, whereas the patient associated with the same verb might be perceived as pitiful. If verbs indeed modify the semantic interpretation of nouns within a phrase, the verb semantics would not only define the action of an event but could indirectly contribute to the representations of the corresponding entities involved.

#### Context & salience affect similarity judgments

Within our study we cannot distinguish an a priori verb bias from other factors such as valence or context-specific effects such as salience. We specifically chose contrasting verbs that could either express a positive, communicative event or a negative, physical contact event. This difference in valence might have made the verb semantic information more salient as compared to that of the nouns. Additionally, in the context of the larger stimulus set, verbs could be broadly divided into only two categories, whereas nouns were more varied, stemming from four distinct semantic categories. The fact that there were fewer semantic categories for verbs may have added to their overall salience. Note, however, that the temporal adverbs could also be categorized into two groups, namely those referring to the same day and those referring to the day before. This binary categorization can be observed in the similarity patterns of factor three. In addition, each individual adverb appeared more frequently than each individual verb, potentially increasing salience for the temporal adverbs. Nonetheless, adverbs did not drive the similarity ratings as much as the verbs did. The overall pattern of the data hence speaks to a more fundamental attentional bias towards the verb beyond simple saliency due to stimulus distribution across semantic categories.

Alternative models of similarity have been developed to address the effects of salience as well as other supposed shortcoming of the geometric approach such as the assumption of symmetry (distance(A,B) = distance(B,A)), which has been disconfirmed in empirical data (Tversky, 1977). As a solution, Tversky suggested a set-theoretic model, within which similarity between two items is a function of the set of their shared features and the two respective sets of their distinctive features. This approach allows context to modulate how strongly certain features are activated and as a consequence influence similarity, accounting for salience effects. For example, when adding a single item to a set of items, which varies in a specific feature, that so far had been shared by all items. The addition of the new variance in that feature will increase perceived similarity of all original items.

It is correct that geometric models per definition impose certain assumptions such as symmetry onto similarity relations. Similarity judgments collected throughout multiple arrangements, however, are not completely incompatible with observations of asymmetry and salience effects. In fact, the repeated sampling of subsets of the total stimulus set assumes the existence of a multitude of conceptual spaces, some more and some less salient, under which similarity can be defined. Under this assumption, distance from A to B might differ from distance from B to A, if the order of comparison evokes different similarity spaces (Decock & Douven, 2011). When combining similarity judgments across subset arrangements those subtle differences get lost and the principle of symmetry will be enforced. Although the final similarity matrix cannot explicitly speak to the multiple underlying dimensions anymore, we have shown that they can nonetheless be extracted through data-driven factorization. The same holds true for more or less salient features within a stimulus set.

#### Neural processing of relational information

It is still an open question how our brains process and represent relational information such as who is the agent or the patient of an event. Many studies that rely on sentence similarity structure to investigate the neural processing of thematic role information, have modeled the similarity structure as a binary metric, distinguishing between item pairs being either maximally similar or not similar at all e.g., Frankland and Greene (2020a); Wang et al. (2017). We have shown that it is possible to retrieve continuous quantitative measures of relational stimuli from empirical data, even in the presence of bias, through perceived similarity judgement tasks. These quantitative models can be compared against brain activity using multivariate analysis techniques (Kriegeskorte et al., 2008). Representational models of individual words have already advanced our understanding of the neurobiology of semantics. For example, through explicitly modeling word semantics, researchers could not only confirm a crucial role for the anterior temporal lobe (in line with previous patient data) but also identify additional frontal and parietal brain areas to be sensitive to modality-independent semantic representations (Bruffaerts et al., 2019). Further, similarity judgments of images have been shown to capture perceptual representations (Hebart et al., 2020) that correlate with multivariate neural representations and have been used to study the temporal dynamics of object recognition in the brain (Cichy et al., 2019).

### Quantifying similarity through vector space models

An alternative approach to quantify semantic representations is based on distributional semantics. Distributional semantics rely on the idea that associated words cooccur in similar contexts. Consequently, given a large variety of contexts, we can find a function that maps each word onto a high-dimensional numerical vector (embedding) capturing a word’s association to all other words in the vocabulary. Recently, artificial neural networks (ANNs) have become prominent for generating word embeddings as a byproduct of unsupervised learning tasks. ANNs are usually trained to predict a word based on its preceding or surrounding context and require large linguistic corpus data for training. The most recent generation of ANNs (e.g., Vaswani et al. (2017); Devlin et al. (2018); Radford et al. (2019)) is able to capture contextualized word meanings, taking into account local sentence context. For example, they will assign distinct vector representations to the word “bank” if preceded by either “river” or “money”. This newest generation of algorithms excels at multiple natural language processing tasks such as text generation, translation, question answering, and cloze tasks (Brown et al., 2020).

Due to their broad success at language tasks, ANNs have caught the interest of language scientists not only as tools for natural language processing but also as mechanistic models for the brain’s language processing. Indeed, several research groups have shown that internal representations in ANNs, emerging during training, predict brain activity above chance (Pereira et al., 2018; Mitchell et al., 2008; Abnar et al., 2019; Schrimpf et al., 2021; Toneva et al., 2020). Specifically, those models implementing attention mechanisms (Vaswani et al., 2017), like the GPT2 model, seem to outperform other architectures (Schrimpf et al., 2021). At the same time, several researchers have raised the criticism that ANNs cannot capture structural relational meaning (Gershman & Tenenbaum, 2015; Puebla et al., 2021). This is an empirical question so that we need focused test-sets, that explicitly probe those semantic dimensions impacting human behavior in order to evaluate what representations these models are learning and how similar they are to neural representations in humans.

We propose that similarity judgments sensitive to relational information might provide a meaningful benchmark for evaluating ANNs as models for human language processing. Previously, ANNs’ have been evaluated not only in terms of their ability to predict brain signals but also with respect to human linguistic behavior such as for example reading times and gaze duration (Schrimpf et al., 2021; Van Schijndel & Linzen, 2018; Merkx & Frank, 2020), and there is a long history of that (e.g., McClelland and Rumelhart 1981). Measures of perceived similarity can provide an additional benchmark of human behavior against which computational models could be evaluated. This approach of evaluating computational models based on human similarity ratings has already proven to deliver insights with respect to ANNs trained on visual object recognition (Jozwik et al., 2017; Peterson et al., 2017). For example, researchers are starting to identify which parameters of the model architecture (e.g., layer depth) are crucial for learning human-like representations (Jozwik et al., 2017). We have shown that similarity judgments are useful tools to investigate representational content of stimuli containing relational features such as thematic roles, even if perception is biased towards single meaning dimensions. Similarity judgments could further serve as a point of comparison for models of human language processing, specifically with respect to emerging relational knowledge. As an example, we compared contextualized embeddings of our stimuli from three state-of-the-art ANNs: GPT2, BERT and its extension, SBERT (Reimers & Gurevych, 2020b). While all ANN models seemed to capture information about thematic roles to some degree, none of them reproduced the verb bias we observed in our behavioral data. This could suggest that current out-of-the-shelf word prediction ANNs might not exploit information in the same way humans do. Whether these ANNs are unable to capture human biases needs to be further tested, however, with a larger behavioral dataset and carefully controlled stimuli to exclude saliency effects of local context. Of course, a comparison with human behavioral data cannot speak to the predictive performance and utility of ANNs as engineering solutions for language tasks. Instead, similarity judgments provide a benchmark to evaluate the ANNs’ utility as mechanistic models of human sentence processing irrespective of their highly successful application as chatbots or for machine translation.

A strength of artificial language models is their ability to quickly process large amounts of data. Can the multiple arrangement task be scaled up to generate a matching number of human judgements? In principle, the multiple arrangement task provides a first noisy estimate of all pairwise similarities rather fast, given that all stimulus items are presented within the first display. Every consecutive display provides additional evidence and so the estimates will get more accurate with time. Hence scaling up is in principle possible but whether the task is practically feasible in terms of duration will depend on the required level of detail. In addition, it is desirable that all stimulus items can be observed in full on the initial display in order to identify those dimensions that are common across the entire stimulus set. In the current study, with 48 full sentences as items, we found that in practice the physical screen size limits the number of items that can be placed around the arena without overlap. We therefore restricted the initial display to ten sentences. We observe that given the subsampling procedure proposed by Kriegeskorte and Mur (Kriegeskorte & Mur, 2012) an average of 58 trials (SD = 11.7 trials) per subject are necessary to collect at least one similarity judgement for every single item pair. For reference, the total number of trials completed was 101 on average (SD = 47 trials). Limiting the size of the initial display to be smaller than the full stimulus set hence greatly reduced the paradigm’s efficiency as the subsampling procedure did not strictly optimize for unseen pairs. Beyond limitations due to screen size, human memory constraints only minimally affect the number of items that can be compared in this task, because on each given trial a subject can base their similarity judgements on a selection of the most important dimensions given a trial-specific item context. Even with hundreds of items, the complexity of the comparison can be reduced for example by creating a small number of piles such that all items are assigned to contrasting points along one dimension. In this case, only the summed duration of all drag-and-drop movements will place a maximum constraint on the number of items possible. Many of the mentioned practical limitations in extending the multiple arrangement task may be bypassed, however, by pooling data from multiple subjects. For example, Hebart and colleagues combined pooling approaches with widespread online distribution to collect a large dataset containing similarity ratings for over 1 million images (Hebart et al., 2020).

### Conclusion and outlook

In conclusion, we evaluated the multiple arrangement task combined with matrix factorization as a suitable tool for quantifying integrated meaning representations. The similarity judgments presented here captured multiple dimensions of sentence meaning while also being sensitive to biases in human sentence comprehension. In the future, the multiple arrangement task could be used to collect perceived sentence similarity on a larger scale. Such a database could provide an additional benchmark when evaluating ANNs as models for human language processing.

## Data Availability

All analysis code as well as the datasets collected for the current study are made available on OSF under the following link: https://osf.io/e2jqs/?view_only=2ac4a0719e4c47e4b8c6ac92f50fdae9

## Appendix: Stimuli

**Table 2:** Full set of sentences

Sentence	
Heute Morgen bestärkte der Therapeut einen Sanitäter	
Heute ermunterte der Sanitäter den Pfleger	
Heute Morgen bejubelte der Radiologe den Internisten	

Sentence	
Vorhin ermutigte der Pfleger einen Chirurgen	
Vorhin tröstete der Chirurg den Radiologen	
Vorhin lobte ein Internist den Therapeuten	
Heute Morgen ermunterte der Therapeut den Handwerker	
Heute Morgen lobte der Sanitäter den Mechaniker	
Heute tröstete der Chirurg einen Tischler	
Heute Morgen ermutigte der Internist einen Zimmermann	
Vorhin bestärkte der Pfleger den Klempner	
Vorhin bejubelte der Radiologe den Elektriker	
Am Vormittag bejubelte der Bassist den Pianisten	
Am Vormittag ermutigte der Sänger den Gitarristen	
Gestern Abend ermunterte der Pianist einen Geiger	
Gestern tröstete der Geiger den Sänger	
Gestern bestärkte der Gitarrist einen Musiker	
Gestern Abend lobte der Musiker den Bassisten	
Am Vormittag bestärkte der Bassist einen Läufer	
Am Vormittag lobte der Pianist den Sportler	
Am Vormittag tröstete der Sänger einen Athleten	
Am Vormittag ermunterte der Gitarrist den Fußballer	
Gestern Abend ermutigte der Geiger den Sprinter	
Gestern Abend bejubelte der Musiker einen Boxer	
Heute Morgen schlug der Zimmermann einen Handwerker	
Heute verprügelte der Mechaniker einen Klempner	
Heute stieß der Klempner einen Tischler	
Heute Morgen verscheuchte der Tischler einen Elektriker	
Vorhin schubste der Handwerker den Mechaniker	
Vorhin schüttelte der Elektriker den Zimmermann	
Heute Morgen schubste der Klempner einen Sänger	
Heute Morgen verscheuchte der Mechaniker einen Gitarristen	
Heute stieß der Elektriker den Bassisten	
Vorhin schüttelte der Zimmermann den Pianisten	
Vorhin schlug ein Handwerker den Geiger	
Vorhin verprügelte der Tischler den Musiker	
Am Vormittag schüttelte der Sportler den Radiologen	
Am Vormittag schlug der Läufer den Chirurgen	
Am Vormittag verscheuchte der Athlet einen Internisten	
Gestern Abend schubste der Boxer einen Sanitäter	
Gestern stieß ein Sprinter einen Pfleger	
Gestern Abend verprügelte ein Fußballer einen Therapeuten	
Am Vormittag verscheuchte der Boxer einen Läufer	
Am Vormittag schubste ein Sprinter den Athleten	
Am Vormittag schlug der Fußballer einen Boxer	
Gestern Abend verprügelte der Läufer den Sportler	
Gestern stieß der Sportler den Sprinter	
Gestern Abend schüttelte der Athlet den Fußballer	
